# Importance of coastal primary production in the northern Baltic Sea

**DOI:** 10.1007/s13280-016-0778-5

**Published:** 2016-04-13

**Authors:** Jenny Ask, Owen Rowe, Sonia Brugel, Mårten Strömgren, Pär Byström, Agneta Andersson

**Affiliations:** 1 Department of Ecology and Environmental Science, Umeå University, 901 87 Umeå, Sweden; 2 Division of Microbiology and Biotechnology, Department of Food and Environmental Sciences, Viikki Biocenter 1, University of Helsinki, Helsinki, Finland; 3 Umeå Marine Sciences Centre, 905 70 Hörnefors, Umeå, Sweden

**Keywords:** Benthic primary production, Pelagic primary production, Benthic contribution, Coastal ecosystems, Bothnian Bay, Northern Baltic Sea

## Abstract

In this study, we measured depth-dependent benthic microalgal primary production in a Bothnian Bay estuary to estimate the benthic contribution to total primary production. In addition, we compiled data on benthic microalgal primary production in the entire Baltic Sea. In the estuary, the benthic habitat contributed 17 % to the total annual primary production, and when upscaling our data to the entire Bothnian Bay, the corresponding value was 31 %. This estimated benthic share (31 %) is three times higher compared to past estimates of 10 %. The main reason for this discrepancy is the lack of data regarding benthic primary production in the northern Baltic Sea, but also that past studies overestimated the importance of pelagic primary production by not correcting for system-specific bathymetric variation. Our study thus highlights the importance of benthic communities for the northern Baltic Sea ecosystem in general and for future management strategies and ecosystem studies in particular.

**Electronic supplementary material:**

The online version of this article (doi:10.1007/s13280-016-0778-5) contains supplementary material, which is available to authorized users.

## Introduction

Organic carbon from algal primary production constitutes an important supply of matter for aquatic food webs, being transferred to higher trophic levels directly via grazing or indirectly via the microbial food web (Legendre and Rassoulzadegan 1995). Primary production by algae (autotrophic production) takes place in the water column and on substrates (e.g. rocks and sediments) as long as the conditions for growth are met, i.e. sufficient amounts of light, essential nutrients and inorganic carbon. Aquatic systems driven by autotrophic primary production often exhibit high food web efficiency and support productive food webs (Berglund et al. 2007). In coastal areas, which are among the most productive ecosystems in the world, both benthic (substrate-associated) and pelagic (water column-associated) habitats contribute to total primary production (Borum and Sand-Jensen 1996; Underwood and Kromkamp 1999; Gerbersdorf et al. 2005; Krause-Jensen et al. 2012). Due to high nutrient concentrations in sediments and sufficient light availability, the coastal ecosystems can in fact be dominated by benthic primary production, a phenomenon that has been recognized in several studies worldwide (reviewed in Cahoon 1999; Gazeau et al. 2004).

Benthic primary producers consist of a large variety of organisms including macroalgae, aquatic plants and microalgae. Microalgae grow on all types of substrates such as rocks, logs, sand and soft sediments and they also grow as epiphytes on macroalgae and aquatic plants. While many studies focus on the growth, productivity and global importance of marine vegetation such as macroalgae and seagrasses (Duarte et al. 2005), much of the coastal benthic primary production can be performed by microalgae, i.e. microphytobenthos (Cahoon 1999; Glud et al. 2002). Benthic microalgae are not only important as primary producers, they also provide sediment stability by producing extracellular carbohydrates (de Brouwer et al. 2003), oxygenating (shallow) bottom waters (Granéli and Sundbäck 1986) and preventing nutrient release to the overlaying water column (Sundbäck 1986). Furthermore, it has been shown that benthic microalgae are a highly utilized resource for higher trophic levels in a variety of aquatic ecosystems (Mallin et al. 1992; Middelburg et al. 2000; Moncreiff and Sullivan 2001; Karlsson and Byström 2005; Karlsson et al. 2009; Vander Zanden et al. 2011; Evrard et al. 2012), for instance, the nearshore benthic habitat was disproportionately preferred compared to the pelagic habitat by salmonid predators in a large lake (Hampton et al. 2011). Hence, the contribution of benthic microalgae to total primary production, and to the total pool of organic carbon constituting the energy for higher trophic levels, can be substantial.

The Baltic Sea is a semi-enclosed, brackish water sea with minimal tidal influence. It is the second largest brackish water body in the world with a drainage basin area 4.3 times larger than the sea itself. Due to its shape, size (~415 000 km^2^, HELCOM) and the usually gentle slope of its coast, it has a relatively high ratio of coastal to open water area and is quite shallow [average depth is 65, 68 and 43 m in the Baltic Proper, the Bothnian Sea and the Bothnian Bay, respectively (Voipio 1981)]. This indicates that relatively large areas, especially in the Bothnian Bay, may potentially receive enough light to support significant benthic primary production.

Environmental variables such as salinity, temperature, nutrients and length of productive season increase in a gradient from north to south in the Baltic Sea. The different basins of the Baltic Sea thus have different environmental conditions and prerequisites for the residing organisms. For instance, it is well known that the diversity of macrofauna increases from north to south with mainly marine species in the south and dominantly freshwater species in the north (Elmgren 1984). The same pattern can be observed for phytoplankton and macroalgae, with increasing primary production (Samuelsson et al. 2006) and total biomass (Kautsky 1988), respectively, in a north-to-south gradient. It has been suggested that there is also a slight increase in benthic primary production in a north-to-south gradient, but the benthic contribution to total primary production decreases in the same gradient (Elmgren 1984). For instance, the benthic contribution in coastal areas (0–25 m depth) is about 50, 23 and 12 % in the Bothnian Bay, the Bothnian Sea and the Baltic Proper, respectively (Kautsky 1995; Kautsky and Kautsky 1995), and 10.7, 2.7 and 3.0 %, respectively, on a whole basin scale (Elmgren 1984). However, these estimates are mainly based on data from studies on macroalgae and to some extent microalgae on hard substrates, while the number of studies including benthic microalgae on soft and sandy sediments is low in the Baltic Proper and virtually non-existent in the northern basins. Hence, the above estimates are likely more accurate for the southern Baltic Sea due to macroalgal dominance, but potentially inaccurate and underestimated in the northern regions due to lower macroalgal dominance and a lack of data regarding benthic microalgal production.

In this study, we measured depth-dependent in situ benthic microalgal primary productivity on rocks and on soft sediment over a summer season in a northern Baltic Sea estuary. We also derived a value for total benthic primary production by combining our measured microalgal values with macroalgal values from the literature. The benthic primary production value was then compared with pelagic primary production in order to get a more comprehensive overview of the relative importance of different types of primary production in the northern Baltic Sea. Published data on benthic microalgal primary production in other areas of the Baltic Sea were also compiled to allow for a broad comparison with our field data.

## Materials and methods

### Study area and environmental data

**Fig. 1:**
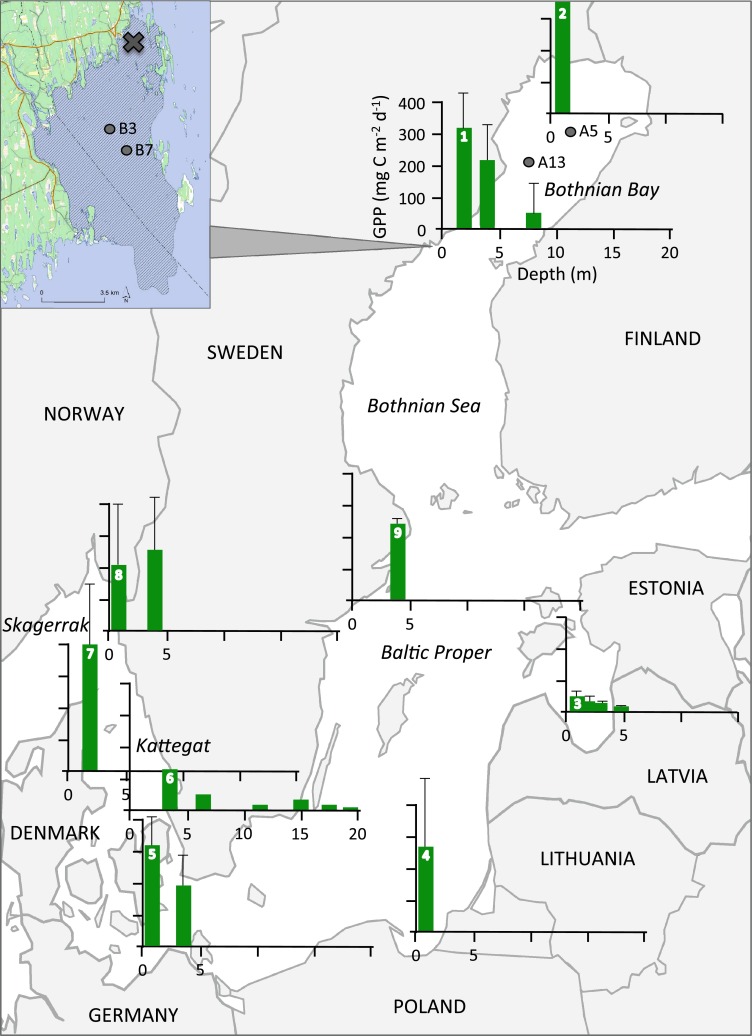
A compilation of studies measuring primary production by benthic microalgae in the Baltic Sea, here divided into five main areas: Bothnian Bay, Bothnian Sea, Baltic Proper, Kattegat and Skagerrak. The insert shows the extension of the Öre estuary (*shaded area*), and the sampling site for this study is marked with an *X*. The monitoring stations used for pelagic primary production are shown in the map (A5 and A13) and in the insert (B3 and B7). For the graphs, benthic gross primary production (GPP, mg C m^−2^ day^−1^) is presented on the *y*-axis and depth (m) on the *x*-axis. All graphs are plotted on the same scale as graph 1 (this study) and arranged so that the bar representing the most shallow depth is placed on or close to the sampling site. *Error bars* represent the standard deviation for a seasonal mean (here March–October), and were calculated when possible. The graphs are numbered (on the bar representing the most shallow depth) and the sources of the data are described accordingly in Table 1

During the 2012 summer season, we quantified primary production on rocks and on soft sediments in the Öre estuary, southern Bothnian Bay (Naturvårdsverket 2007), Sweden (Fig. 1). The sampling site (63°34′47″N 19°51′37″E) was dominated by rocks between 0 and 2 m depth and by soft sediments from 2 m depth onwards, with scattered stands of macrophytes that increased in abundance towards the end of the summer but never reached more than 20–30 % coverage (visual determination). Temperature and photosynthetically active radiation (PAR, 400–700 nm) profiles were recorded using a CTD probe (Seabird 19 Plus V2 SeaCat profiler, Sea-Bird Electronics INC., equipped with a Biospherical QSP-2350L Quantum Scalar PAR sensor) during mid-day in the entire water column (down to 8 m depth) at the deepest point of the sampling site (10 m), which is also where all the samples were incubated (see below). The vertical light attenuation coefficient (*K*_d_) was calculated as the slope of the depth–ln(PAR) linear relationship (Kirk 2011) between 2 and 8 m; PAR values at depths shallower than 2 m were excluded from the regression due to unstable values near the surface. PAR was also calculated for 10 m following the extension of the depth–ln(PAR) relationship described above. The total amount of PAR reaching any given depth [PAR_incubation_*Xm* (mol quanta m^−2^ day^−1^)] during the 24-h benthic incubation period (see below) was calculated according to Eq. 1:

1 $$ {\text{PAR}}_{\text{incubation}}  Xm = \frac{{I_{xm\;t} }}{{I_{0\;t} }} \times \sum {I_{0} } , $$

where *I*_*xm t*_ is the PAR at *Xm* at time *t* (µmol quanta m^−2^ s^−1^), *I*_0 *t*_ is the incident PAR at time *t* (µmol quanta m^−2^ s^−1^) and *∑I*_0_ is the incident PAR summarized over the 24-h benthic incubation period (mol quanta m^−2^ day^−1^).

### Pelagic measurements

Data on daily pelagic primary productivity were obtained from the Swedish national monitoring programme based at Umeå Marine Sciences Centre (Hörnefors, Sweden). The pelagic primary productivity is measured by the ^14^C incubation method as described in the HELCOM Combine Manual, Annex C-5 (HELCOM 2014) and in Wikner and Andersson (2012). Incubation times are short, 2–4 h, and after a +6 % correction term (Gargas 1975) the method should generate primary production values that are close to gross primary production (Gargas 1975; Marra 2009), henceforth referred to as “primary production”.

Respiration was measured in the pelagic habitat by gently filling 120-ml dark glass bottles with water from 0.5, 2, 4 and 8 m depths (*n* = 11 per depth). The bottles were closed with a thick rubber stopper and a metal crimp cap, and two bottles per depth (start samples) were acidified immediately with 1 ml 2 N HCl (Ask et al. 2009b). The acidification stops the biological processes and drives all the carbonate species in the dissolved inorganic carbon (DIC) pool to CO_2_. The remaining bottles were incubated in darkness at ambient temperature in laboratory climate chambers, and by acidifying the water the incubation was terminated after 3, 6 and 10 days (*n* = 3 per depth and day). After acidification, a 50 ml headspace (using N_2_ gas) was created in each bottle using two syringes with attached thin needles as temporary sampling ports. The bottles were shaken for 1 min after which 40 mL of the headspace gas was retrieved and injected into closed, empty vials. The vials were analysed for CO_2_ using a gas chromatography–flame ionization detector (Perkin-Elmer) equipped with a headspace autosampler (GC) and the values were calibrated against reference gases with known concentrations. The daily respiration rate was equal to the slope of the linear change in DIC over time.

### Benthic measurements

Primary productivity and respiration associated with soft sediments were measured on four occasions (end of May, end of June, early August and end of August) by collecting intact sediment cores from three depths (2, 4 and 8 m) using Plexiglas tubes (6.4 cm inner diameter) and a sediment gravity corer (Ask et al. 2009b). The sediment cores collected with the tubes were largely undisturbed (the only, very slight, disturbance was the collection itself) and contained the naturally occurring benthic community, including microalgae and micro-, meio- and sometimes macrofauna. The 12 tubes, containing an approximately 10-cm-high sediment core and 15 cm of overlaying water (corresponding to approximately 0.5 l of water), were sealed airtight and incubated in situ at the depths of collection with or without a dark outer cover (*n*_light_ = *n*_dark_ = 2 per depth). The incubation time was approximately 24 h in order to generate daily values. The tubes were attached to a line hanging freely from an anchored surface buoy at the deepest point of the sampling site (10 m). Start and stop samples of DIC were collected before and after the incubation, respectively, by transferring a portion (4 mL) of the overlaying water to closed vials pre-injected with 100 µL 2 N HCl. The DIC samples were analysed for CO_2_ as described above for pelagic respiration. Respiration (mg C m^−2^ day^−1^) was calculated as the production of DIC in the dark cores over the incubation period (∆DIC_dark_). Since production and consumption of DIC occur simultaneously in the light cores, daily gross primary productivity (GPP, mg C m^−2^ day^−1^) was calculated as the difference between the light and dark cores (GPP = ∆DIC_light_ − ∆DIC_dark_) assuming that light and dark algal respiration rates are equal (Carignan et al. 2000; Williams and Lefevre 2008). This calculation generates negative GPP values (reflecting the consumption of DIC), but the absolute values are presented for clarity and are henceforth referred to as “primary production”. The daily rates of primary production and respiration from the soft sediment measurements were corrected for the area of the tubes and for pelagic primary production and respiration in relation to the water volume overlaying the sediment in the tubes.

For primary productivity and respiration associated with rocks, we placed a number of stone-discs (*n* = 16 per depth, i.e. *n* = 4 per sampling occasion and depth) in open racks attached at 2, 4 and 8 m depths to a line hanging freely from an anchored surface buoy at the deepest point of the sampling site (10 m). The stone-discs were placed just after ice break-up (mid/end of March) in order to allow for algae to colonize. On each sampling occasion (same as for soft-bottom samples), four stone-discs per depth were retrieved, as were four equally sized natural rocks from 0.5 to 1 m depths, for primary productivity and respiration measurements. The algae-colonized rocks and stone-discs were placed in separate Plexiglas tubes, with or without a dark outer cover, filled with water from the sampling depth (approximately 0.8 L of water, *n*_light_ = *n*_dark_ = 2). The tubes were sealed airtight and incubated in situ at the depths of collection and the water was analysed for DIC following the same procedure as described for soft-bottom primary productivity. Daily rates of gross primary production (henceforth “primary production”) and respiration from the hard surface measurements were calculated as above and were corrected for the area of the stones or discs and for pelagic primary production and respiration in relation to the water volume in the tubes.

### Bathymetry and upscaling

In order to generate bathymetric characteristics, depth–area–volume relationships for the Bothnian Bay and for the Öre estuary were calculated from a digital elevation model for the Baltic Sea region with 500 m (Brydsten 2009) and 25 m (Brydsten and Jansson, *unpublished data*) cell size, respectively. The bathymetric relationships were used to calculate a depth–area–volume-weighted mean value for both coastal and whole-system primary production at each sampling occasion. The total area and volume of each studied system (Öre estuary and Bothnian Bay) were divided into depth intervals (0–1, 1–3, 3–6, 6–10 and 10 m–max, see Supplementary material A) that incorporated the sampling depths (0.5, 2, 4, 8 and 15 m), where the 15 m pelagic values were set to represent the 10-m max depth interval even though this overestimates pelagic production. No benthic samples were taken below 10 m and benthic primary production was therefore assumed to be zero in the 10-m max depth interval. The extension of the coastal zone was estimated from the digital elevation model (above) and the maximum depth at which 1 % of surface PAR was remaining (calculated from our own data, indicating the proportion of illuminated benthic and pelagic habitats).

The bottom substrate in the Öre estuary and the Bothnian Bay was coarsely divided into soft sediment, rocks and sand following the results from intensive monitoring efforts in Sweden (EU Interreg IVA-funded projects “ULTRA” and “SUPERB”, County Administrative Board of Västerbotten) and Finland (The Finnish Inventory Program for the Underwater Marine Environment (VELMU)). Data on macroalgal coverage were derived from the County Administrative Board of Västerbotten, Sweden (EU Interreg IVA-funded projects “ULTRA” and “SUPERB”, County Administrative Board of Västerbotten), and microalgae were assumed to cover the area not covered by macroalgae. Data for microalgal primary production on soft sediments and rocks (hard substrates) were derived from our own study, whereas data for microalgal primary production on sand (343 mg C m^−2^ day^−1^ at approximately 0.5 m) were derived from Kautsky and Foberg (2001). Since no data are available on depth-dependent primary production on sand in the area, we assumed that it decreases with depth in the same way as primary production on soft sediments (Fig. 1). Data on macroalgal and macrophyte primary production (503 mg C m^−2^ day^−1^ at approximately 0.5 m and 200 mg C m^−2^ day^−1^ at approximately 4 m) were derived from Kautsky and Foberg (2001) and Jansson and Wulff (1977).

To calculate the area-weighted benthic mean primary production value, the area of each depth interval was divided into substrate classes and each substrate class was divided into a macroalgal or microalgal share generating a number of sub-areas. The sub-areas were each multiplied by the respective measured or compiled daily primary production value and subsequently summarized to cover the total area of the depth interval. The total benthic primary production values representing each depth interval were summarized and divided by the total area of the system. The bathymetric relationships were also used to calculate a volume- and area-weighted mean value for pelagic primary production that is comparable to the benthic, i.e. the total volume of the respective interval was multiplied by the pelagic primary production value representing that depth interval. The values were then summarized and divided by the total area of the system.

A mean value for benthic and pelagic primary production during the productive season was calculated by averaging the values from the four sampling occasions, but in order to allow for comparisons with other studies we also calculated an annual mean value. However, a model relating our benthic primary production values to PAR (and other environmental parameters) on a yearly basis was not possible due to few sampling occasions. Instead, the yearly value was obtained by first assigning the measured values to a summer month (May, June, July and August, respectively) and then by assuming negligible production during the four winter months (November–February). Benthic primary production values for March and April were obtained by linear extrapolation between zero (February) and the first sampling occasion (May). Benthic primary production values for September and October were obtained by linear extrapolation between the last sampling occasion (August) and zero (November). The production values representing each month were multiplied by the number of days and the yearly value could thus be calculated by summarizing the monthly values. The benthic primary production during November, December, January and February was assumed to be negligible due to the few hours of sunlight, the low angle of incoming light and the more or less permanent ice-cover in the Bothnian Bay. Data from the Swedish national monitoring programme at Umeå Marine Sciences Centre also show that the PAR values are below the detection limit during this period (Siv Huseby, Umeå Marine Sciences Centre, *personal communication*). Nevertheless, this assumption is likely to generate an underestimated annual benthic primary production value since significant benthic primary production has been recorded during winter in polar areas (Attard et al. 2014). Pelagic primary production values are available for the entire year (Swedish national monitoring programme); thus, averaged daily values representing each month could be multiplied with the number of days for the respective month and subsequently summarized to obtain the annual value.

### Literature survey

In addition to the field study, we searched the literature database (ISI journals) for field studies presenting data on primary production by microalgae in benthic habitats (i.e. microphytobenthos) in the Baltic Sea (Table 1). Our focus was to compile data from as many locations as possible in order to get a wide geographical distribution of benthic primary production. Where multiple studies exist from the same area, we chose the one that measured benthic production at the greatest depth (e.g. Sundbäck and Jönsson 1988; Sundbäck et al. 2004). We also aimed to compile data from as many types of substrates as possible (i.e. sand, soft sediment, rocks); however, due to the lack of data regarding the primary production of epiphytic microalgae (i.e. microalgae growing on macrophytes or aquatic plants), these important benthic primary producers are not included. In two of the studies (Meyercordt and Meyer-Reil 1999; Urban-Malinga and Wiktor 2003), only hourly values were presented. We multiplied the hourly values by the day length for the given area and time of year in order to obtain daily values (mg C m^−2^ day^−1^). This calculation might however slightly overestimate the production values since incubations were done during mid-day when irradiance generally is at its maximum, or underestimate the values if the algal communities are experiencing photoinhibition. For seasonal studies, we calculated an average for the productive season (here: March–October).

**Table 1:** Information regarding the literature data compiled for the review part of this study. The methods are described using four categories: a. how the primary productivity values were measured; b. where (in situ or lab) and for how long the samples were incubated; c. how daily values were obtained (if given); and d. if the sediment/bottom substrate were undisturbed (“intact”) or disturbed (“slurry”). Data from the studies denoted with * are used in the calculations of the coastal and whole-system production in the Öre estuary and Bothnian Bay

ID	Location	Substrate	Depths (m)	Method (a–d)	Source
1	Bothnian Bay	Soft sediment and rocks	2, 4, 8	a. DIC; b. in situ, 24 h; c. incubation time; d. intact	This study*
2	Bothnian Bay	Sand	0.5–1	a. O_2_; b. in situ, 2–4 h; c. light factor; d. intact	Kautsky and Foberg (2001)*
3	Baltic Proper	Sandy sediments	0.5, 1, 2, 2.5, 3, 5	a. ^14^C; b. in situ, 2–3 h; c. insolation values; d. intact	Vilbaste et al. (2000)
4	Baltic Proper	Sand	0.5	a. O_2_; b. in situ, 4 h; c. not given; d. slurry	Urban-Malinga and Wiktor (2003)
5	Baltic Proper	Soft mud/sand	0.6, 3.4	a. O_2_; b. in situ, 4–5 h; c. not given; d. intact	Meyercordt and Meyer-Reil (1999)
6	Kattegat	Sand/silt	3.5, 6.5, 11.5, 15, 17.5, 19.5	a. ^14^C; b. lab, 2 h; c. P-I curves; d. slurry	Sundbäck and Jönsson (1988)
7	Kattegat	Soft mud/silt	2	a. O_2_; b. in situ, 3–6 h; c. daylight duration; d. intact	Gazeau et al. (2005)
8	Skagerrak	Sand	0.5, 4	a. ^14^C; b. in situ, 2–3 h; c. insolation values; d. intact	Sundbäck et al. (1996)
9	Baltic Proper	Soft sediments	4	a. O_2_; b. in situ, 24 h; c. incubation time; d. intact	Jansson and Wulff (1977)*

While pelagic primary productivity often is measured by the ^14^C incorporation technique, there is no standard technique for measuring benthic primary productivity. Most studies compiled here used slightly different methods, such as measuring the ^14^C uptake or measuring the changes in dissolved oxygen (O_2_) or gaseous carbon dioxide (CO_2_) concentrations (Table 1). The techniques were applied to intact sediment cores (Vilbaste et al. 2000; Ask et al. 2009b) or to sediment slurries (Jönsson 1991) that were incubated in situ or in the lab, using different versions of the light–dark chamber method (Howarth and Michaels 2000). A comparison between the ^14^C incorporation technique and the CO_2_ method (Ask et al. 2009b), and between the CO_2_ and O_2_ techniques (Kristensen 1993), for benthic microalgal primary productivity measurements indicate that comparing the data is possible. Furthermore, great care was taken when compiling the data to only choose studies applying methods currently in use and that were deemed to be comparable. However, comparing data derived from studies using different techniques is never problem free. Thus, the data for the literature survey in this study should mainly be looked upon as an overview indicative of large-scale patterns.

## Results

Most studies found in the literature survey encompassing estimates of benthic microalgal primary production from the Baltic Sea were performed on sites dominated by sandy substrates, were focusing on shallow areas (often <5 m) and were mainly conducted in the southern basins (Fig. 1; Table 1). Unlike pelagic primary production, which increases from north to south (Samuelsson et al. 2006), there appears to be no clear trend in benthic microalgal primary production (Fig. 1). In fact, benthic microalgal production seems to be approximately equal in the southern and northern parts of the Baltic Sea (Fig. 1).

**Fig. 2:**
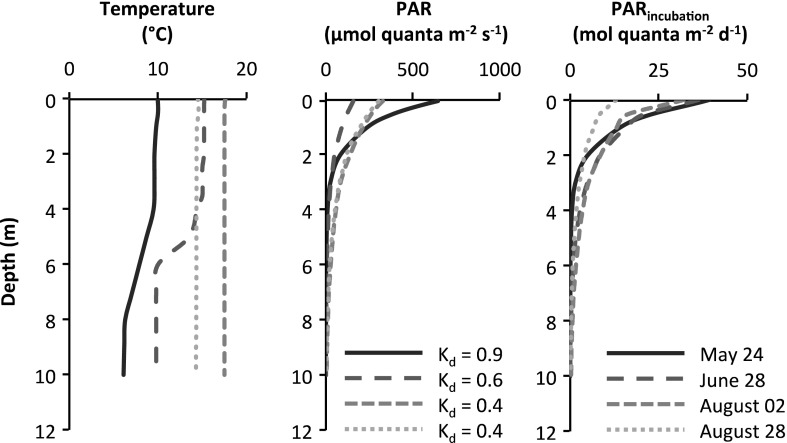
Profiles for temperature, photosynthetically active radiation (PAR) and the cumulative amount of PAR during the benthic incubation period (24 h, PAR_incubation_) for the different sampling occasions. The vertical light attenuation coefficient (*K*_d_, m^−1^) was calculated as the slope of the depth–ln(PAR) linear relationship between 2 and 8 m. PAR at 10 m was also calculated from the extension of this relationship (i.e. not measured)

**Fig. 3:**
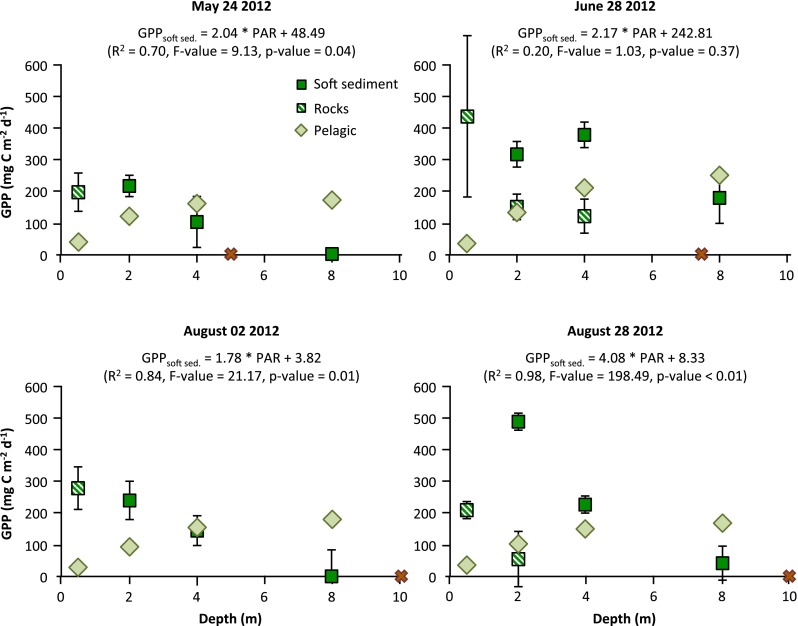
Benthic gross primary production (GPP) on soft sediment and rocks, and pelagic GPP, measured on four occasions during the 2012 summer season in the Öre estuary, Umeå, Sweden. The benthic values are the un-treated measured values, whereas the pelagic values are depth-integrated, and *error bars* (not always visible) for the benthic samples represent the standard deviation based on two replicates. The depth at which 1 % of surface PAR remains is marked with a *red X*. The linear relationship between soft sediment GPP (GPPsoft sed.) and PAR is given for each sampling occasion, however, the low number of replicates for these relationships (and for the benthic sample *error bars*) should be noted

For the estuary study, PAR values decreased with depth with only slightly different light attenuation coefficients at each sampling occasion (Fig. 2). The cumulative amount of PAR during the benthic incubation period decreased with depth in a similar manner as seen in the PAR profile, although the internal relationship between the sampling dates differ due to longer daylight periods especially in May and June (Fig. 2). The depth at which 1 % of surface PAR remained was 5.1, 7.6, 10.3 and 10.2 m (Fig. 3). Since 1 % of the light could reach a depth of at least 10 m, we defined the coastal zone in the Öre estuary and Bothnian Bay as the area (and volume) between the shoreline and 10 m of depth (Supplementary material A). This resulted in that at least 34.1 and 26.9 % of the Öre estuary and Bothnian Bay area, respectively (Table 2), provide suitable light conditions for benthic primary production. Of the total water volume in the Öre estuary and in the Bothnian Bay, 53.4 and 23.0 %, respectively, is found between 0 and 10 m (Table 2), whereas only 2.7 and 10.9 %, respectively, of the total volume is found in the coastal zone (0–10 m). Temperature decreased with depth on the two first sampling occasions with a possible thermocline between 4 and 8 m (Fig. 2), whereas it was relatively stable on the remaining sampling occasions.

**Table 2:** Total and relative (in %) volumes (m^3^) and areas (m^2^) of the different depth intervals in the Öre estuary, Sweden, and Bothnian Bay derived from the digital elevation model (see “Materials and methods” section)

Depth interval (m)	Öre estuary				Bothnian Bay			
	Volume	Area	Volume	Area	Volume	Area	Volume	Area
	(m^3^)	(m^2^)	(%)	(%)	(m^3^)	(m^2^)	(%)	(%)
0–1	6.5E+07	2.6E+06	6.3	4.0	3.6E+10	3.1E+09	2.7	8.6
1–3	1.2E+08	4.2E+06	12.0	6.4	6.5E+10	1.8E+09	4.9	5.0
3–6	1.7E+08	7.0E+06	16.3	10.5	9.1E+10	2.4E+09	6.9	6.5
6–10	1.9E+08	8.7E+06	18.8	13.2	1.1E+11	2.5E+09	8.5	6.8
10–max	4.8E+08	4.4E+07	46.6	65.9	1.0E+12	2.7E+10	77.0	73.1
0–10	5.5E+08	2.3E+07	53.4	34.1	3.0E+11	9.8E+09	23.0	26.9
**Total**	**1.0E+09**	**6.6E+07**	**100**	**100**	**1.3E+12**	**3.6E+10**	**100**	**100**

Benthic primary production by microalgae on soft sediments in the Öre estuary was measurable at all sampling depths and decreased with depth on all sampling occasions (Fig. 3, but see also Fig. 1 for a seasonal mean) largely consistent with the decreasing PAR values (Fig. 3). Soft sediment estimates were more than twice as high compared to pelagic estimates of primary production at 2 m, similar at 4 m, but lower at 8 m (Fig. 3). Unfortunately, some sampling points are missing for the primary production measurements on rocks (Fig. 3) since we lost many stone-discs due to inclement weather. Primary production on rocks (when applicable) was always lower than that on soft sediments, and was of the same magnitude as pelagic primary production already at 2 m depths (Fig. 3). Primary production values on rocks at 0.5 m were in the same range as those of soft sediment primary production at 2 m, except in late August when it was lower (Fig. 3).

At the most shallow depth interval (0–1 m), the distribution between the three bottom substrate classes used in this study was quite equal both in the Öre estuary and in the Bothnian Bay (Table 3). The amount of hard substrate increased slightly with depth, whereas the soft and sandy substrates decreased (Table 3). The macroalgal coverage decreased from 25 % at 0–2 m to 1 % at 10 m on soft and sandy bottoms, and from 5 to 0.5 % on hard bottoms (Table 3). Macroalgal cover was only available from Sweden but was assumed to be valid also for the Finnish coastline.

**Table 3:** Bottom surface area of the study systems was coarsely divided into three substrate classes: “hard” (gravel, stones, boulders and base rock), “soft” (silt, clay and mud) and “sand”. The average proportion of these substrate classes in the different depth intervals used in this study was derived from the Swedish (EU Interreg IVA-funded projects “ULTRA” and “SUPERB”, County Administrative Board of Västerbotten) and Finnish [The Finnish Inventory Program for the Underwater Marine Environment (VELMU)] monitoring programmes. An average of the Swedish and Finnish data was used for the Bothnian Bay, whereas only the data from Sweden were used for the Öre estuary. Macroalgal cover was only available from Sweden (EU Interreg IVA-funded projects “ULTRA” and “SUPERB”, County Administrative Board of Västerbotten), but is assumed to be valid also for the Finnish coastline. There are a few percent of the Finnish bottom surface area that are “unclassified” (not shown)

Depth interval (m)	Bottom substrate, Sweden			Bottom substrate, Finland			Macroalgal cover	
	Hard	Soft	Sand	Hard	Soft	Sand	Soft and sand	Hard
	(%)	(%)	(%)	(%)	(%)	(%)	(%)	(%)
0–1	39.2	29.4	31.4	25.5	33.9	36.1	24.9	4.4
1–3	46.0	25.7	28.3	42.1	27.0	29.8	17.2	3.5
3–6	60.5	20.1	19.4	52.9	23.6	22.8	5.1	1.3
6–10	68.1	19.5	12.4	53.6	28.0	17.8	0.8	0.2

The area-weighted (i.e. the bathymetry of the system is taken into account) mean seasonal value for benthic primary production in the Öre estuary was 133.2 mg C m^−2^ day^−1^ in the coastal zone (0–10 m) and 45.4 mg C m^−2^ day^−1^ for the whole estuary (Table 4). On an annual basis, these values were 23.6 and 8.0 g C m^−2^ year^−1^, respectively (Table 4). When upscaling the benthic values to the level of the entire Bothnian Bay, the values are slightly higher in the Bothnian Bay compared to the Öre estuary (Table 4), highlighting the bathymetric differences between the systems (Table 2). Volume- and area-weighted mean values for pelagic primary production were similar to benthic primary production in the coastal areas, but higher on a whole-system scale (Table 4), resulting in a benthic share of total production between 43 and 65 % on a coastal scale and between 17 and 31 % at the whole-system scale in the studied systems (Table 4). The microalgal share of total benthic primary production (microalgal + macroalgal) was 86 % in the Öre estuary and 80 % in the Bothnian Bay.

**Table 4:** Area–depth–volume-weighted seasonal and yearly mean values of benthic and pelagic gross primary production (GPP in mg C m^−2^ day^−1^ and g C m^−2^ year^−1^, respectively) in the Öre estuary and Bothnian Bay for 2012. The benthic share of total primary production (benthic + pelagic) is shown by “% benthic”. The area between the shoreline and 10 m depths defines the coastal area, and the coastal volume is the volume related to this area (Supplementary material A). The difference in benthic contribution between the estuary and the Bothnian Bay mainly depends on bathymetric differences

Site	Average summer GPP (mg C m^−2^ day^−1^)				Yearly GPP (g C m^−2^ year^−1^)			
	Coastal		Whole system		Coastal		Whole system	
	Benthic	Pelagic	Benthic	Pelagic	Benthic	Pelagic	Benthic	Pelagic
Öre estuary	133.2	136.0	45.4	163.8	23.6	31.9	8.0	38.6
% benthic	49		22		43		17	
Bothnian Bay	196.5	107.2	52.9	123.1	36.9	25.1	9.4	21.3
% benthic	65		30		59		31	

## Discussion

In this study, we measured primary productivity by microalgae growing on rocks and soft sediments in the northern Baltic Sea, and the values were in the same order of magnitude as those from clear-water lakes where the total primary production can be completely dominated by benthic primary producers (Ask et al. 2009b). Our values are also in correspondence with estimates from coastal areas in the southern Baltic Sea (Sundbäck and Jönsson 1988; Sundbäck et al. 1996; Meyercordt and Meyer-Reil 1999; Vilbaste et al. 2000; Urban-Malinga and Wiktor 2003; Sundbäck et al. 2004; Gazeau et al. 2005) and thus add to the growing awareness regarding the importance of benthic primary producers in coastal areas on a global scale (Pinckney and Zingmark 1993; MacIntyre et al. 1996; Cahoon 1999; Underwood and Kromkamp 1999; Glud et al. 2002; Gattuso et al. 2006; Glud et al. 2009; Attard et al. 2014).

Benthic primary production (i.e. by both micro- and macroalgae) has rarely been quantified in the northern Baltic Sea (but see Kautsky et al. 1981; Elmgren 1984; Kautsky and Kautsky 1995; Kautsky and Foberg 2001) and data for microalgal primary production are in fact sparse in the entire Baltic Sea (Fig. 1). For instance, Elmgren (1984) suggested that benthic primary production was 20 g C m^−2^ year^−1^ in the littoral zone down to 10 m depths in the Bothnian Bay based on estimates from Kautsky et al. (1981). This is slightly lower than our benthic primary production estimates of 23.6 and 36.9 g C m^−2^ year^−1^ in the coastal zone down to 10 m depths in the Öre estuary and in the Bothnian Bay, respectively (Table 4). Our upscaled annual benthic primary production estimate for the entire Bothnian Bay was 9.4 g C m^−2^ year^−1^ (Table 4), which is about three times higher than the 3 g C m^−2^ year^−1^ suggested in Elmgren (1984). Furthermore, when comparing benthic with pelagic values on a whole-system scale, we estimated the benthic share of total primary production to be 31 % on a yearly basis in the Bothnian Bay, which is also three times higher than previously estimated (Elmgren 1984). It should be noted that these types of whole-system estimates are often based on data from different studies and on a relatively low number of studied sites, suggesting that they are subjected to uncertainties. However, whole-system estimates are still valuable since they facilitate broader scale comparisons.

Hence, the difference in the estimates presented here most likely relates to the lack of comprehensive benthic data (Fig. 1) and proper bathymetric relationships, but to some extent also to the commonly used assumptions on how pelagic values are upscaled to production estimates on whole basin scales. Most, if not all, studies presenting values on pelagic primary production in the different basins of the Baltic Sea give depth-integrated (trapezoid integration, often 0–20 m) mean values (Elmgren 1984; Wasmund et al. 2001; Samuelsson et al. 2006; Larsson et al. 2010). This depth-integrated approach does not take the bathymetry of the specific system into account, which does not pose a problem when only comparing site-specific pelagic production, as long as all sites compared are 20 m or deeper (i.e. if the primary production value is depth integrated between 0 and 20 m). However, this may pose problems when estimating the total carbon budget of a system, or when relating pelagic to benthic primary production, since the depth integration (when extended to whole systems) incorporates pelagic volumes in coastal areas that do not exist. When comparing our volume- and area-weighted pelagic values to pelagic values calculated with the commonly used depth-integrated approach (i.e. same input data but the bathymetry was not taken into account), the pelagic values were about 39 and 13 % smaller on a coastal and a whole-system scale, respectively. This calculation resulted in a 12 and 3 % lower benthic share estimate, respectively. This implies a shortcoming in the way total pelagic primary production is generally estimated, especially when comparing pelagic and benthic contributions and their relative importance for ecosystem processes, and also in attempts to calculate carbon budgets and upscaling data to whole-ecosystem production estimates.

The PAR profiles and total amount of PAR during the incubation period differed between the sampling dates (Fig. 2), with the highest benthic primary production values measured during the day of lowest PAR values (Figs. 2, 3). Although the depth-dependent benthic primary production was highly related to the PAR values at any given date (except on June 28, Fig. 3), other parameters such as temperature, algal biomass, algal community composition and grazing pressure will also be important over larger temporal scales. The possible drivers of benthic primary production are not evaluated further in this study since the main objective was to investigate the relative importance of pelagic and benthic primary production. Primary production in general is highly dependent on light, indicating that low or high incident PAR at the sampling date should not affect the benthic/pelagic relationship too much.

Despite the very low levels of light at 8 m depths in the study area (Fig. 2), we found measurable and significant rates of benthic primary productivity (Fig. 3). This supports previous findings and assumptions regarding benthic/attached algae as being highly able to adapt to low light conditions (Cahoon 1999; Wulff et al. 2005; Gomez et al. 2009), more so than pelagic algae. The amount of light needed for benthic algal growth has also been shown to be temperature dependent (Hancke et al. 2014), indicating that algae in cold areas or during winter can sustain growth at even lower light intensities (Glud et al. 2002; Gomez et al. 2009; Attard et al. 2014). In this study, we used the depth (10 m) at which at least 1 % of surface PAR remains (often referred to as the photic zone) to determine the extension of the coastal zone. However, primary production is not a function of the relative amount of PAR, but of the total amount reaching a certain depth. Hence, if it is assumed, as it often is, that no primary production takes place beneath the photic zone, then estimations of total primary production might be significantly underestimated, especially when taking low-light adapted benthic algae into account. Since no measurements were taken beneath 8 m, the possible underestimation of benthic primary production could not be fully evaluated in this study, and thus poses an important challenge for future studies.

Benthic primary producers are often defined as being everything from macroalgae and aquatic plants to microalgae growing on a variety of substrates. This grouping is problematic since the benthic primary producers differ in their growth, photosynthetic ability, nutrient and substrate requirements and more importantly in their role in the food web. Microalgae are readily grazed and constitute an important energetic base for higher trophic levels (Karlsson and Byström 2005; Karlsson et al. 2009) via meio- (Sundbäck et al. 1996) and macrofauna (Cahoon 1999; Evrard et al. 2012), whereas macroalgae and plants, although grazed to some extent (Duarte and Cebrian 1996), are more important for providing structure and shelter for higher trophic level organisms (Schindler and Scheuerell 2002). Furthermore, the microalgae growing on soft sediments will have higher productivity rates than those growing on rocks or other hard substrates since the soft sediment microalgae have ready access to nutrients stored in the sediment (Vadeboncoeur et al. 2006), while microalgae on hard substrates generally acquire their nutrients from the surrounding water and thus have to compete with phytoplankton, especially in nutrient-poor systems such as the northern Baltic Sea (Andersson et al. 1996; 2015). This is also indicated in this study with generally lower rates of primary productivity on rocks compared to soft sediments at the same depths (Fig. 3). The estimated microalgal share of the total benthic primary production was 86 % in the Öre estuary and 80 % in the Bothnian Bay emphasizing the importance of microalgae for benthic primary production estimates. However, we have not taken the very commonly occurring microalgae growing as epiphytes on macroalga and macrophytes into account (Johansson et al. 2012; Albertsson 2014), which then likely underestimates the importance of benthic microalgae and also benthic primary production in general. For future studies regarding productivity, food web structure and trophic transfer efficiency in aquatic ecosystems, detailed coastal maps determining bottom substrates and sediment type in a depth gradient, combined with knowledge regarding the distribution and relative importance of primary production between different habitats (i.e. benthic vs. pelagic) as well as between growth forms (i.e. micro- vs. macroalgae), are therefore crucial.

The shallow and sheltered coastal areas provide important spawning and nursing habitats for both benthic and pelagic fish in marine systems (Snickars et al. 2005; Eriksson et al. 2011; Polte et al. 2014; Sundblad et al. 2014). Higher temperatures compared to open waters, both promoting high production (food resources) and facilitating rapid development and growth of juvenile fish, together with structural refuges from predation, have been suggested to be the main reasons behind their importance (Bohling et al. 1991; Gibson et al. 1998; Veneranta et al. 2011, 2013; Polte et al. 2014). Moreover, ontogenetic shifts from feeding on small-sized zooplankton to larger sized benthic prey are common, and benthic prey may constitute a large fraction of the diets in both benthivorous and piscivorous fish in the Baltic Sea (Hansson et al. 1997; Mustamäki et al. 2014). Benthic prey have also been shown to be an essential resource for intermediate size stages of piscivores (Persson and De Roos 2012; van Leeuwen et al. 2013), and piscivores are suggested to be keystone species for the structure and function of both coastal and offshore ecosystems in the Baltic Sea (Casini et al. 2009; Eriksson et al. 2011; van Leeuwen et al. 2013). Thus, the highly productive benthic areas studied here may contribute to piscivore densities to a relatively large extent and consequently to their important structuring role of Baltic Sea ecosystem functions and services.

Shallow coastal areas are recognized as being highly valuable ecosystems and many sites along the Swedish coast are being classified as “Natura 2000” areas (Naturvårdsverket 2003). However, the classifications are often based on criteria regarding the amount of vegetation (i.e. macrophytes and macroalgae), and biomass of macrofauna and fish in the area. Microalgae (except for phytoplankton) are usually not taken into consideration (Naturvårdsverket 2007), although a high coverage of microalgae on hard substrates and as epiphytes on macroalgae are being observed in the northern parts of the Baltic Sea (Kautsky and Kautsky 1995; Johansson et al. 2012; Albertsson 2014). Compared to pelagic systems in the Baltic Sea, the benthic habitat currently receives little research effort and monitoring attention, with the exception of sedimentation records, heavily polluted sites and areas of increasing anoxia. However, in this study we show that primary production by soft-bottom benthic microalgae (alone) in the northern parts of the Baltic Sea reaches values as high as those in the southern Baltic Sea and that the benthic contribution to total basin-scale primary production is significant (31 %). Moreover, we present arguments and data suggesting that the share of benthic production to total ecosystem production may have been underestimated in previous studies due to lack of data and simplified assumptions when upscaling pelagic primary production to whole-ecosystem estimates. Based on the results from our study, it is thus evident that the very sparse amount of data regarding the magnitude, productivity and depth distribution of primary production by different types of benthic algae constitute a critical gap in our knowledge regarding Baltic Sea ecosystem productivity, especially in the light of expected effects of future climate change. Climate change will have multiple effects on the natural environment and the effects will be both direct, such as increasing temperatures, and indirect, such as an increased input of organic matter (carbon and nutrients) to aquatic ecosystems. Since the dissolved organic carbon from terrestrial environments often is colored, it may have large effects on the productivity of the recipient waters via light attenuation (Ask et al. 2009a; Wikner and Andersson 2012; Dupont and Aksnes 2013; Lefebure et al. 2013). In fact, it has been shown that the negative effects of light attenuation on primary producers, mainly benthic microalgae, may cascade all the way up the food web and decrease fish biomass and production (Karlsson et al. 2009). From the findings in this study, and regarding local (i.e. dredging, urban development) and global (i.e. climate change) environmental stressors affecting coastal habitats, we argue that it is of utmost importance that the role of especially benthic microalgal communities is considered in future ecosystem studies and in the development of future management strategies for the Baltic Sea ecosystems.

## Electronic supplementary material

Below is the link to the electronic supplementary material.

Supplementary material 1 (PDF 65 kb)
