# Supplementary material for: Importance of coastal primary production in the northern Baltic Sea
Source: Ambio. 2016 Apr 13;45(6):635–48. doi: 10.1007/s13280-016-0778-5 (PMC5012998; doi:10.1007/s13280-016-0778-5)

AMBIO

Electronic Supplementary Material
This supplementary material has not been peer reviewed.

Title: **Importance of coastal primary production in the northern Baltic Sea**

Authors: Jenny Ask, Owen Rowe, Sonia Brugel, Mårten Strömgren, Pär Byström, Agneta Andersson

Supplementary material A

A schematic drawing of the volume and area divisions used in this manuscript. The grey region represents the total volume between 0-10 m, divided into the specific depth intervals. The dotted grey region represents the coastal volume and the coastal area. The benthic (0.5m, 2m, 4m and 8m) and pelagic (0.5m, 2m, 4m, 8m and 15m) sampling depths are shown as dots.

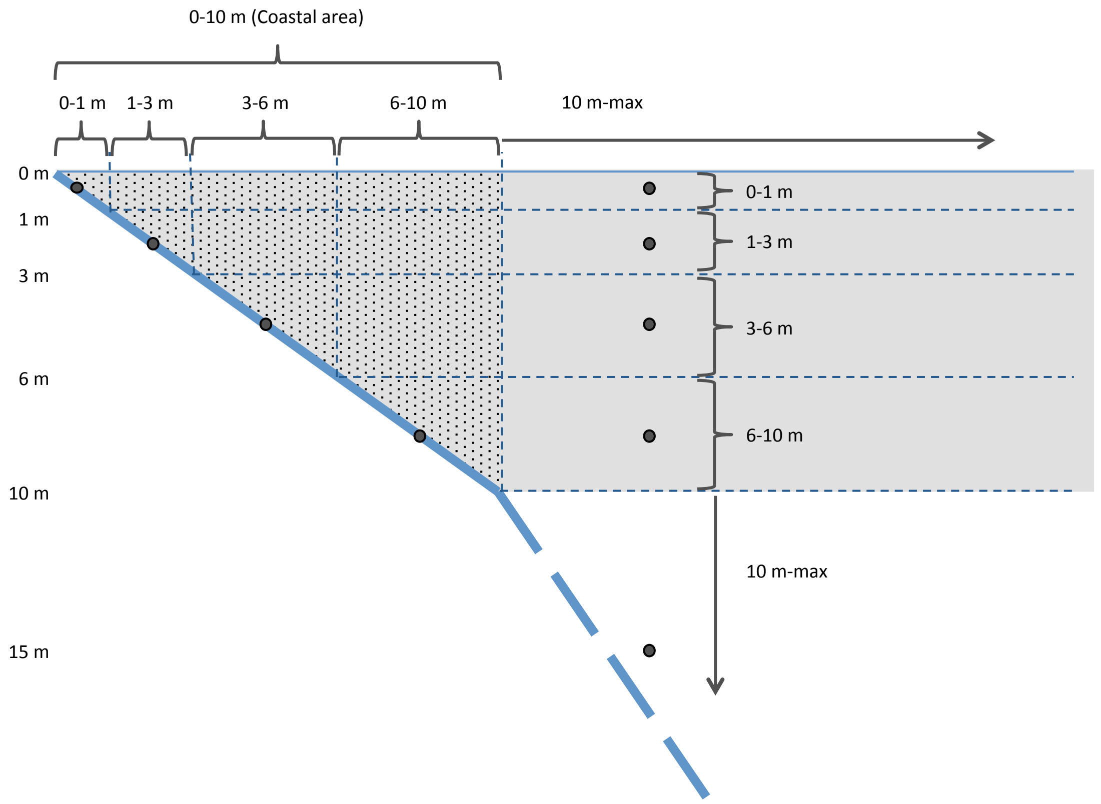

Supplement: Supplementary file 1 — Supplementary material 1 (PDF 65 kb) [file 13280_2016_778_MOESM1_ESM.pdf]
